# Supplementary material for: Adaptation to the cervical environment is associated with increased antibiotic susceptibility in Neisseria gonorrhoeae
Source: Nat Commun. 2020 Aug 17;11:4126. doi: 10.1038/s41467-020-17980-1 (PMC7431566; doi:10.1038/s41467-020-17980-1)
Supplement: Supplementary file 1 — Supplementary Information [file 41467_2020_17980_MOESM1_ESM.pdf]

Adaptation to the cervical environment is associated with increased antibiotic susceptibility in *Neisseria gonorrhoeae* (Ma and Mortimer et al.)

Supplementary Information

Supplementary Tables 3
Supplementary Table 1. Datasets included in global meta-analysis collection. 3
Supplementary Table 2. Restoration of the *mtrC* coding frame in a clinical isolate by transformation increases MICs to azithromycin, ceftriaxone, and ciprofloxacin. 4
Supplementary Table 3. Prevalence of predicted LOF mutations in efflux pump genes for both global and Australian datasets. 5
Supplementary Table 4. Association between sexual behavior and mtrC LOF in global dataset. 6
Supplementary Table 5. Association between site of infection and mtrC LOF in global dataset. 6
Supplementary Table 6. Association between site of infection and *mtrC* LOF in validation dataset. 6
Supplementary Table 7. Association between sexual behavior and *mtrC* LOF in validation dataset. 6
Supplementary Table 8. *mtrA* LOF occurs most often in genetic backgrounds without *mtrR* or *mtr* promoter mutations in global dataset. 7
Supplementary Table 9. Association between site of infection and *mtrA* LOF in global dataset 7
Supplementary Table 10. Association between sexual behavior and mtrA LOF in global dataset 7
Supplementary Table 11. *mtrA* LOF occurs most often in genetic backgrounds with *mtrR* or *mtr* promoter mutations in validation dataset. 8
Supplementary Table 13. *mtrC* LOF occurs most often in genetic backgrounds with *mtrR* or *mtr* promoter mutations in validation dataset. 8
Supplementary Table 14. Association between site of infection and *farA* LOF in global dataset. 9
Supplementary Table 15. Association between sexual behavior and *farA* LOF in global dataset. 9
Supplementary Table 16. Association between site of infection and farA LOF in validation dataset. 9
Supplementary Table 17. Association between sexual behavior and farA LOF in validation dataset. 9

Supplementary Figures 10
Supplementary Figure 1. Diagnostic Q-Q plots of expected versus observed p-values for GWAS on a) azithromycin, b) ceftriaxone, and c) ciprofloxacin. 10
Supplementary Figure 2. Alignment of nucleotide sequences for strains with representative LOF mutations observed in a) *mtrC*, b) *mtrA*, and c) *farA* in the global dataset......................11
Supplementary Figure 3. Phylogenetic distribution of gonococcal *mtrC*, *mtrA*, and *farA* LOF alleles with patient site of infection (n=2742) in global dataset. 12
Supplementary Figure 4. MIC distributions for isolates with indicated resistance determinants for azithromycin, ceftriaxone, and ciprofloxacin respectively, stratified by *mtrC* genotypic status...13
Supplementary Figure 5. Gonococcal *mtrC* and *farA* LOF mutations are associated with sexual behavior and site of infection in the validation dataset. 14
Supplementary Figure 6. Phylogenetic distribution of gonococcal *mtrC* LOF alleles with patient site of infection (n=2186) in the validation dataset..15
Supplementary References 16

Supplementary Tables

Supplementary Table 1. Datasets included in global meta-analysis collection. All isolates that passed genomics quality control filters (see methods) with associated azithromycin, or ceftriaxone, or ciprofloxacin metadata were included (n=4852 in total). Euro-GASP = European Gonococcal Antimicrobial Surveillance Program; CDC GISP = Centers for Disease Control and Prevention Gonococcal Isolate Surveillance Project.

**Publication**	**Study summary**	**Timespan**	**Included number**
Mortimer et al., 2020[1]	Transmission and AMR surveillance in New York City, USA	2011-2015	888
Sánchez-Busó et al., 2019[2]	Worldwide phylogeography and evolution of gonococcus	1979-2012	408
Yahara et al., 2018[3]	AMR surveillance in Kyoto and Osaka, Japan	1996-2015	260
Ryan et al., 2018[4]	AMR surveillance in Ireland	2012-2016	39
Harris et al., 2018[5]	Genomic survey across European Euro-GASP participant countries (n=20)	2013	1048
Fifer et al., 2018[6]	High-level azithromycin resistance outbreak in UK	2004-2017	50
Lee et al., 2018[7]	Genomic epidemiology in New Zealand	2014-2015	397
Kwong et al., 2017[8]	Transmission among MSM in Melbourne, Australia	2005-2014	94
Eyre et al., 2017[9] and De Silva et al., 2016[10]	Transmission in Brighton, UK	2004-2011	231
Grad et al., 2016[11] and 2014[12]	AMR surveillance across CDC GISP clinics, USA	2000-2013	1100
Demczuk et al., 2016[13]	Azithromycin resistance surveillance in Canada	1991-2014	199
Demczuk et al., 2015[14]	Cephalosporin decreased susceptibility surveillance in Canada	1989-2013	114
Ezewudo et al., 2015[15]	Population structure and AMR surveillance	1982-2011	54

Supplementary Table 2. Restoration of the *mtrC* coding frame in a clinical isolate by transformation increases MICs to azithromycin, ceftriaxone, and ciprofloxacin. MIC Etests were conducted in triplicate with all results reported. Statistical significance for MIC differences between parental strain and transformant strain was assessed by a two-sample t-test after setting the value of "≥ 32" to 32.

**Strain**	**Azithromycin**	**Ceftriaxone**	**Ciprofloxacin**
NY0195	0.064, 0.094, 0.094	0.016, 0.023, 0.023	6, 6, 4
NY0195 *mtrC*(in-frame)	0.5, 0.75, 0.75	0.064, 0.064, 0.064	≥ 32, ≥ 32, ≥ 32
	$p = 0.0187$	$p = 0.00289$	$p = 0.000624$

Supplementary Table 3. Prevalence of predicted LOF mutations in efflux pump genes for both global and Australian datasets. Counts for *mtrA* LOF mutations in the absence of other epistatic *mtrCDE* overexpression mutations are listed first, with counts for total number of *mtrA* LOF mutations regardless of *mtrCDE* overexpression status listed in parentheses.

**Dataset**	**Gene**	**N LOF**	**N Total**	**Percentage**
Global	*farA*	332	4838	6.86%
Global	*farB*	2	4850	0.04%
Global	*norM*	2	4852	0.04%
Global	*macA*	1	4847	0.02%
Global	*macB*	13	4845	0.27%
Global	*mtrR*	386	4845	7.97%
Global	*mtrA*	268 (362)	4842	5.45% (7.48%)
Global	*mtrC*	185	4847	3.82%
Global	*mtrD*	10	4815	0.21%
Global	*mtrE*	0	4849	0.00%
Australia	*farA*	225	2180	10.32%
Australia	*farB*	1	2186	0.05%
Australia	*norM*	0	2186	0.00%
Australia	*macA*	0	2186	0.00%
Australia	*macB*	0	2186	0.00%
Australia	*mtrA*	4 (85)	2186	0.18% (3.89%)
Australia	*mtrR*	253	2183	11.59%
Australia	*mtrC*	35	2186	1.60%
Australia	*mtrD*	2	2185	0.09%
Australia	*mtrE*	0	2186	0.00%

Supplementary Table 4. Association between sexual behavior and mtrC LOF in global dataset. *mtrC* LOF is significantly associated with sexual behavior (p = 0.04021, Two- Sided Fisher's Exact Test for Count Data).

mtrC	**WSM**	**MSW**	**MSMW**	**MSM**
Intact	30	598	123	1158
LOF	3	28	4	31

Supplementary Table 5. Association between site of infection and mtrC LOF in global dataset. *mtrC* LOF is significantly associated with site of infection ($p = 6.487 \times 10^{-5}$, Two- Sided Fisher's Exact Test for Count Data).

mtrC	**Cervix**	**Pharynx**	**Rectum**	**Urethra**
Intact	113	103	242	2167
LOF	16	3	4	82

Supplementary Table 6. Association between site of infection and *mtrC* LOF in validation dataset. *mtrC* LOF is significantly associated with site of infection (p = 0.02561, Two- Sided Fisher's Exact Test for Count Data).

mtrC	**Cervix**	**Pharynx**	**Rectum**	**Urethra**
Intact	218	383	625	867
LOF	9	3	7	15

Supplementary Table 7. Association between sexual behavior and *mtrC* LOF in validation dataset. *mtrC* LOF is significantly associated with sexual behavior (p = 0.01803, Two- Sided Fisher's Exact Test for Count Data).

mtrC	**WSM**	**MSW**	**MSM**
Intact	268	243	1424
LOF	10	4	17

Supplementary Table 8. *mtrA* LOF occurs most often in genetic backgrounds without *mtrR* or *mtr* promoter mutations in global dataset.

***mtrCDE* upregulation background**	***mtrA***	**n**
Yes	Intact	4113
No	Intact	355
Yes	LOF	89
No	LOF	268
Unknown	Intact	52
Unknown	LOF	5

Supplementary Table 9. Association between site of infection and *mtrA* LOF in global dataset. *mtrA* LOF is significantly associated with site of infection ($p = 1.626 \times 10^{-12}$, Two- Sided Fisher's Exact Test for Count Data).

mtrA	**Cervix**	**Pharynx**	**Rectum**	**Urethra**
Intact	104	102	242	2187
LOF	25	4	4	61

Supplementary Table 10. Association between sexual behavior and mtrA LOF in global dataset. *mtrA* LOF is significantly associated with sexual behavior ($p = 1.805 \times 10^{-11}$, Two-Sided Fisher's Exact Test for Count Data).

mtrA	**WSM**	**MSW**	**MSMW**	**MSM**
Intact	27	598	127	1181
LOF	6	28	0	7

Supplementary Table 11. ***mtrA*** **LOF occurs most often in genetic backgrounds with** ***mtrR*** **or** ***mtr*** **promoter mutations in validation dataset.**

mtrCDE **upregulation background**	***mtrA***	**n**
Yes	Intact	2012
No	Intact	87
Yes	LOF	81
No	LOF	4
Unknown	Intact	2

Supplementary Table 12. ***mtrC*** **LOF occurs most often in genetic backgrounds with** ***mtrR*** **or** ***mtr*** **promoter mutations in global dataset.**

mtrCDE **upregulation background**	***mtrC***	**n**
Yes	Intact	4028
No	Intact	617
Yes	LOF	174
No	LOF	6
Unknown	Intact	52
Unknown	LOF	5

Supplementary Table 13. ***mtrC*** **LOF occurs most often in genetic backgrounds with** ***mtrR*** **or** ***mtr*** **promoter mutations in validation dataset.**

mtrCDE **upregulation background**	***mtrC***	**N**
Yes	Intact	2060
No	Intact	89
Yes	LOF	33
No	LOF	2
Unknown	Intact	2

Supplementary Table 14. Association between site of infection and *farA* LOF in global dataset. *mtrA* LOF is significantly associated with site of infection ($p = 1.775 \times 10^{-12}$, Two- Sided Fisher's Exact Test for Count Data).

farA	**Cervix**	**Pharynx**	**Rectum**	**Urethra**
Intact	96	103	234	2129
LOF	33	3	12	117

Supplementary Table 15. Association between sexual behavior and *farA* LOF in global dataset. *mtrA* LOF is significantly associated with sexual behavior ($p = 5.055 \times 10^{-10}$, Two-Sided Fisher's Exact Test for Count Data).

farA	**WSM**	**MSW**	**MSMW**	**MSM**
Intact	25	585	125	1166
LOF	8	40	2	22

Supplementary Table 16. Association between site of infection and farA LOF in validation dataset. *mtrA* LOF is significantly associated with site of infection ($p < 2.2 \times 10^{-16}$, Two- Sided Fisher's Exact Test for Count Data).

farA	**Cervix**	**Pharynx**	**Rectum**	**Urethra**
Intact	160	354	609	782
LOF	67	31	21	98

Supplementary Table 17. Association between sexual behavior and farA LOF in validation dataset. *mtrA* LOF is significantly associated with sexual behavior ($p < 2.2 \times 10^{-16}$, Two- Sided Fisher's Exact Test for Count Data).

farA	**WSM**	**MSW**	**MSM**
Intact	197	186	1386
LOF	81	60	51

Supplementary Figures

a)

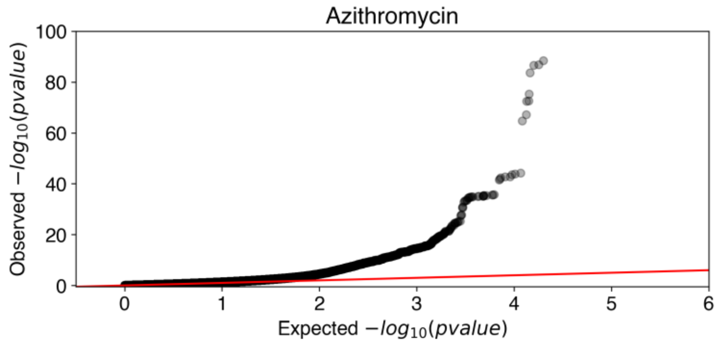

b)

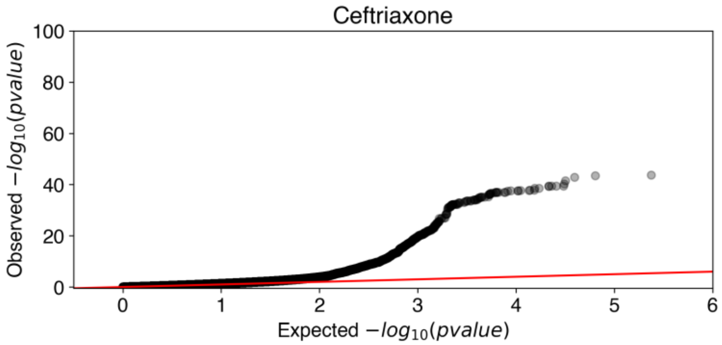

c)

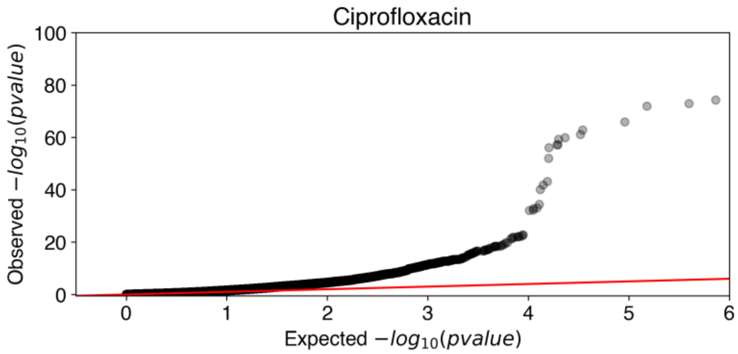

Supplementary Figure 1. Diagnostic Q-Q plots of expected versus observed p-values for GWAS on a) azithromycin, b) ceftriaxone, and c) ciprofloxacin. In the absence of confounders such as population structure, *p*-values are distributed uniformly and would be expected to lie along the y=x line (in red) before diverging at higher -$\log_{10}$(*p*-values) due to true causal variants[16]. Q-Q plots for all three antibiotics appear to be well-behaved, indicating that the steps we have taken to control for population structure (i.e., using a linear mixed model parameterized by the recombination-corrected phylogeny) were adequate. Highly significant markers corresponding to diverging variants at higher -$\log_{10}$(*p*-values) were confirmed to map to known causal variants for all three antibiotics (see Supplementary Table 3).

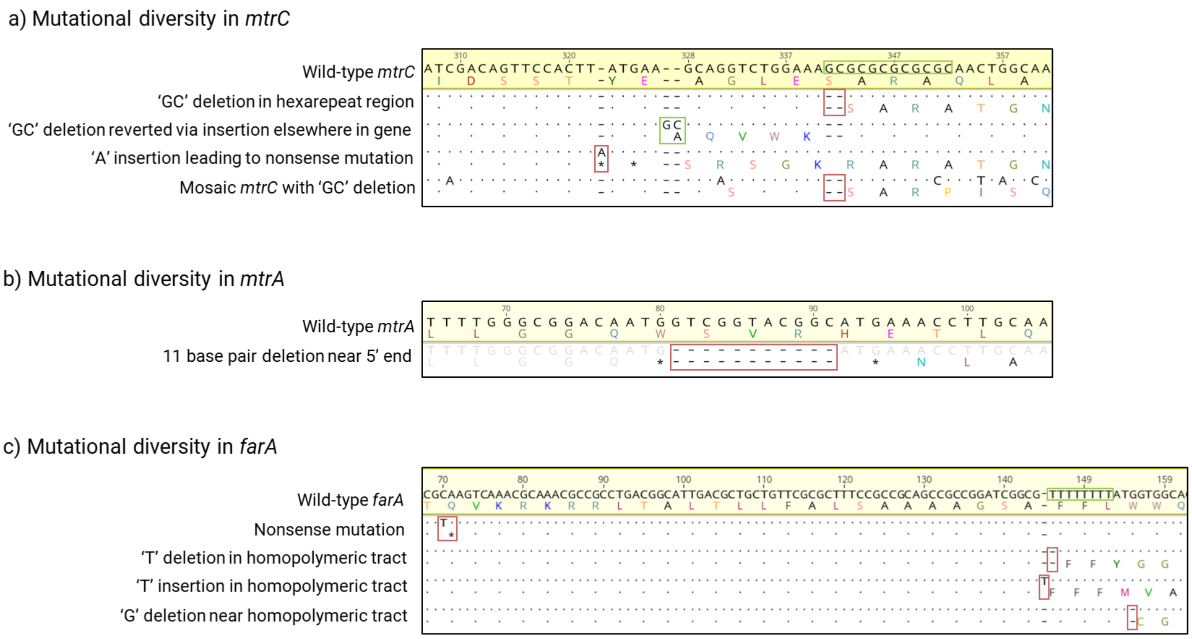

Supplementary Figure 2. Alignment of nucleotide sequences for strains with representative LOF mutations observed in a) *mtrC*, b) *mtrA*, and c) *farA* in the global dataset. The wild-type reference sequences (FA1090 for *mtrC* and *farA*, FA19 for *mtrA*) are shown at the top of the alignment highlighted in yellow. Nucleotide sequences were depicted in black with the corresponding amino acid translations directly under. Dots in LOF sequences represent exact match to the wild-type reference sequence. For *mtrC*, the hexarepeat tract was boxed in the reference genome in green, and mutations leading to LOFs were boxed in red. For *mtrA*, the 11-bp deletion leading to *mtrA* LOF was boxed in red. For *farA*, the repeat tract of Ts was boxed in green, and mutations leading to LOFs were boxed in red. All alignments were visualized in Geneious Prime (see methods).

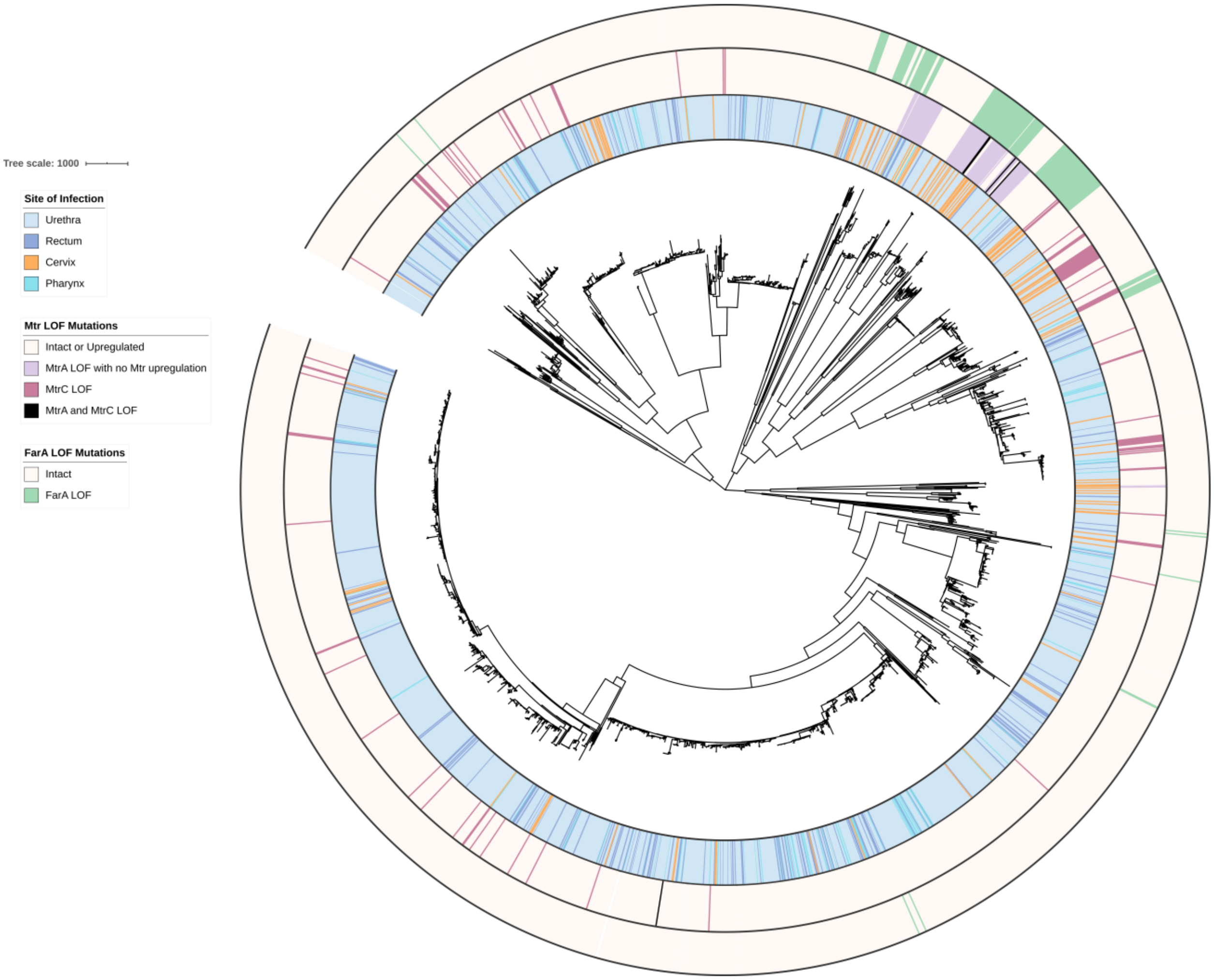

Supplementary Figure 3. Phylogenetic distribution of gonococcal *mtrC*, *mtrA*, and *farA* LOF alleles with patient site of infection (n=2742) in global dataset. The recombination-corrected maximum likelihood phylogeny based on 36347 SNPs is shown annotated with rings (from innermost to outermost) for site of infection, *mtrCDE* regulon LOF mutations, and *farA* LOF mutations. Branch length represents total number of substitutions after removal of predicted recombination.

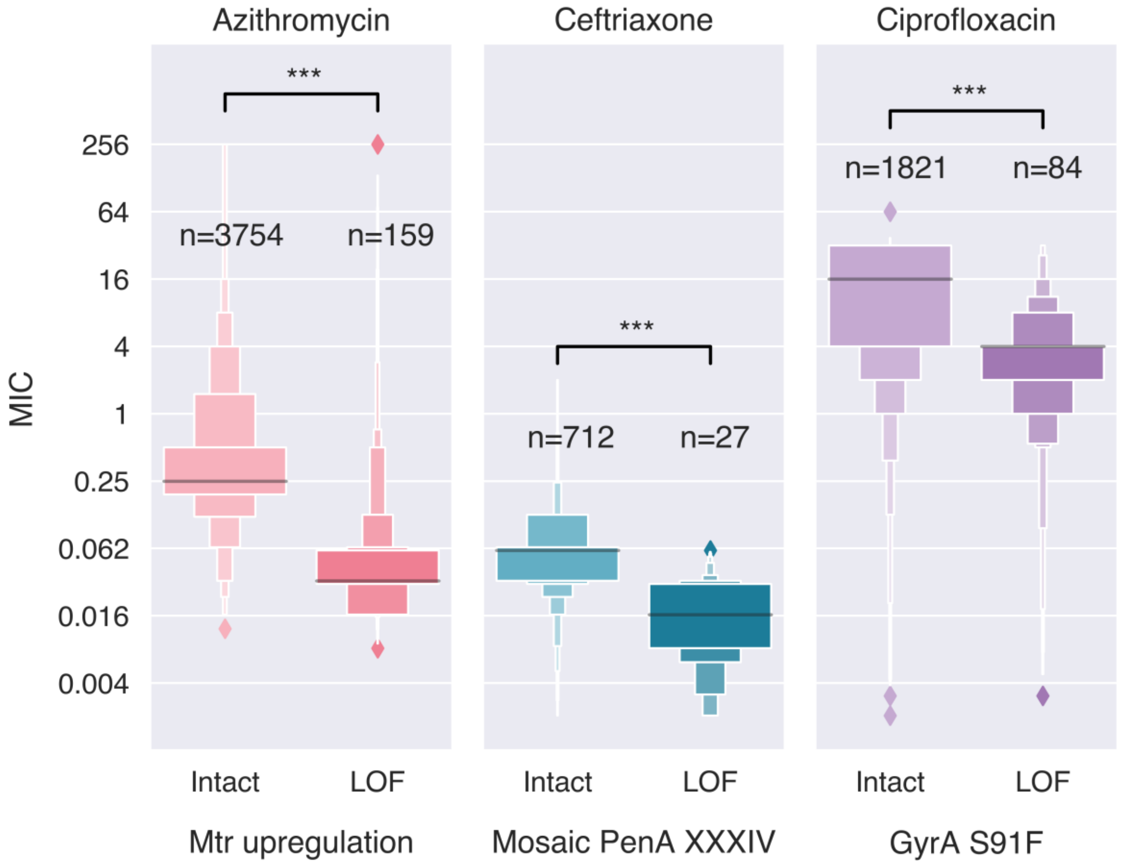

Supplementary Figure 4. MIC distributions for isolates with indicated resistance determinants for azithromycin, ceftriaxone, and ciprofloxacin respectively, stratified by *mtrC* genotypic status. Statistical significance between *mtrC* intact and LOF MIC distributions was assessed by Mann-Whitney U Test: * p<0.05, ** p<0.01, and *** p<0.001. Enhanced box plots (also known as letter-value plots[17]) were drawn using default settings in the Python Seaborn visualization package (version 0.9.0). Interpretation differs from standard box-and-whisker plots: the central half of the MIC distribution is within the widest box; the central three-quarters of the distribution is within the widest and second widest box; the central three-eighths of the distribution is within the widest, second widest, and third widest box; and so on. The proportion of outliers was set to the default 0.007, and the number of boxes depicted is selected proportionally with respect to the total number of samples.

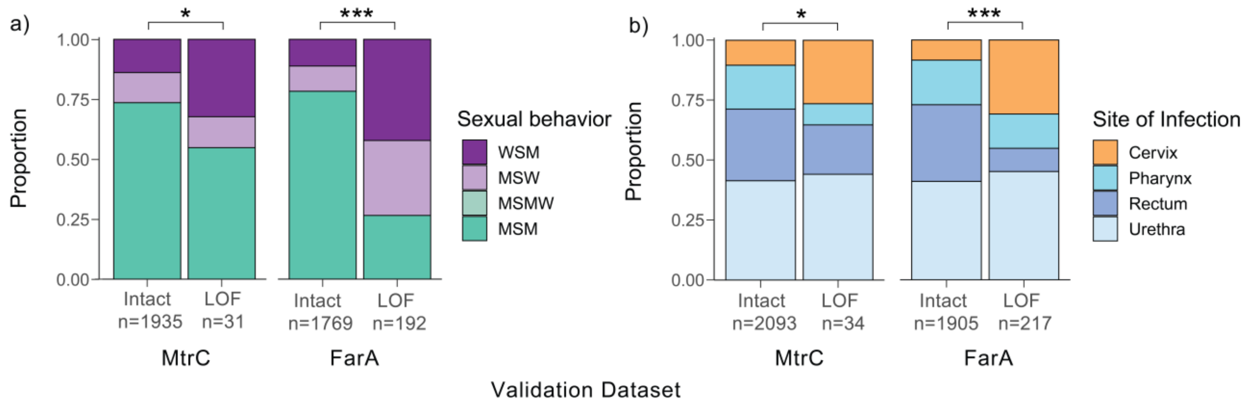

Supplementary Figure 5. Gonococcal *mtrC* and *farA* LOF mutations are associated with sexual behavior and site of infection in the validation dataset. a) Sex partner information in patients infected with isolates with either intact or LOF alleles of *mtrC* (left) or *farA* (right). MSMW patients were labelled as MSM in the validation dataset[18]. b) Site of infection in patients infected with isolates with either intact or LOF alleles of *mtrC* (left) or *farA* (right) datasets. Statistical significance between intact versus LOF patient metadata distributions was assessed by Fisher's exact test: * $p<0.05$, ** $p<0.01$, and *** $p<0.001$. Exact *p*-values from left to right for analyses conducted in a) were 0.0180 and $< 2.20\times10^{-16}$, and for b) were 0.0256 and $< 2.20\times10^{-16}$. WSM = women who have sex with men, MSW = men who have sex with women, MSMW = men who have sex with men and women, MSM = men who have sex with men.

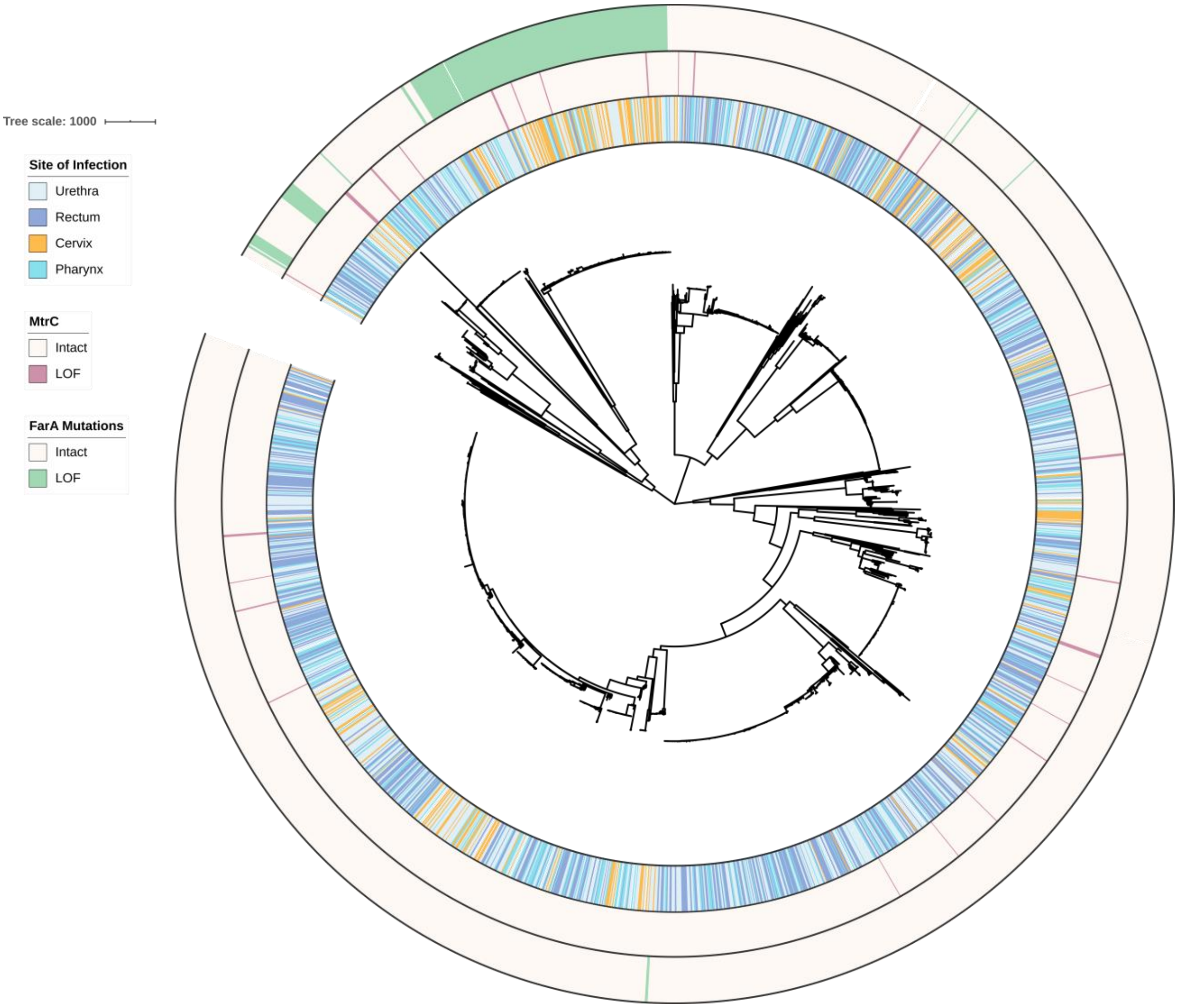

Supplementary Figure 6. Phylogenetic distribution of gonococcal *mtrC* LOF alleles with patient site of infection (n=2186) in the validation dataset. The recombination-corrected maximum likelihood phylogeny based on 26669 SNPs is shown annotated with rings (from innermost to outermost) for site of infection, *mtrC* LOF mutations, and *farA* LOF mutations. Branch length represents total number of substitutions after removal of predicted recombination.

Supplementary References

1 Mortimer, T. D. *et al.* The distribution and spread of susceptible and resistant *Neisseria gonorrhoeae* across demographic groups in a major metropolitan center. Preprint at https://www.medrxiv.org/content/10.1101/2020.04.30.20086413v1 (2020).

2 Sanchez-Buso, L. *et al.* The impact of antimicrobials on gonococcal evolution. *Nat Microbiol*, doi:10.1038/s41564-019-0501-y (2019).

3 Yahara, K. *et al.* Genomic surveillance of Neisseria gonorrhoeae to investigate the distribution and evolution of antimicrobial-resistance determinants and lineages. *Microb Genom* **4**, doi:10.1099/mgen.0.000205 (2018).

4 Ryan, L. *et al.* Antimicrobial resistance and molecular epidemiology using whole-genome sequencing of Neisseria gonorrhoeae in Ireland, 2014-2016: focus on extended-spectrum cephalosporins and azithromycin. *Eur J Clin Microbiol Infect Dis*, doi:10.1007/s10096-018-3296-5 (2018).

5 Harris, S. R. *et al.* Public health surveillance of multidrug-resistant clones of Neisseria gonorrhoeae in Europe: a genomic survey. *Lancet Infect Dis* **18**, 758-768, doi:10.1016/S1473-3099(18)30225-1 (2018).

6 Fifer, H. *et al.* Sustained transmission of high-level azithromycin-resistant Neisseria gonorrhoeae in England: an observational study. *Lancet Infect Dis* **18**, 573-581, doi:10.1016/S1473-3099(18)30122-1 (2018).

7 Lee, R. S. *et al.* Genomic epidemiology and antimicrobial resistance of Neisseria gonorrhoeae in New Zealand. *J Antimicrob Chemother* **73**, 353-364, doi:10.1093/jac/dkx405 (2018).

8 Kwong, J. C. *et al.* Whole-genome sequencing reveals transmission of gonococcal antibiotic resistance among men who have sex with men: an observational study. *Sex Transm Infect* **94**, 151-157, doi:10.1136/sextrans-2017-053287 (2018).

9 Eyre, D. W. *et al.* WGS to predict antibiotic MICs for Neisseria gonorrhoeae. *J Antimicrob Chemother* **72**, 1937-1947, doi:10.1093/jac/dkx067 (2017).

10 De Silva, D. *et al.* Whole-genome sequencing to determine transmission of Neisseria gonorrhoeae: an observational study. *Lancet Infect Dis* **16**, 1295-1303, doi:10.1016/S1473-3099(16)30157-8 (2016).

11 Grad, Y. H. *et al.* Genomic Epidemiology of Gonococcal Resistance to Extended-Spectrum Cephalosporins, Macrolides, and Fluoroquinolones in the United States, 2000-2013. *J Infect Dis* **214**, 1579-1587, doi:10.1093/infdis/jiw420 (2016).

12 Grad, Y. H. *et al.* Genomic epidemiology of Neisseria gonorrhoeae with reduced susceptibility to cefixime in the USA: a retrospective observational study. *Lancet Infect Dis* **14**, 220-226, doi:10.1016/S1473-3099(13)70693-5 (2014).

13 Demczuk, W. *et al.* Genomic Epidemiology and Molecular Resistance Mechanisms of Azithromycin-Resistant Neisseria gonorrhoeae in Canada from 1997 to 2014. *J Clin Microbiol* **54**, 1304-1313, doi:10.1128/JCM.03195-15 (2016).

14 Demczuk, W. *et al.* Whole-genome phylogenomic heterogeneity of Neisseria gonorrhoeae isolates with decreased cephalosporin susceptibility collected in Canada

between 1989 and 2013. *J Clin Microbiol* **53**, 191-200, doi:10.1128/JCM.02589-14 (2015).

15 Ezewudo, M. N. *et al.* Population structure of Neisseria gonorrhoeae based on whole genome data and its relationship with antibiotic resistance. *PeerJ* **3**, e806, doi:10.7717/peerj.806 (2015).

16 Power, R. A., Parkhill, J. & de Oliveira, T. Microbial genome-wide association studies: lessons from human GWAS. *Nat Rev Genet* **18**, 41-50, doi:10.1038/nrg.2016.132 (2017).

17 Hofmann, H., Wickham, H. & Kafadar, K. Letter-Value Plots: Boxplots for Large Data. *J Comput Graph Stat* **26**, 469-477, doi:10.1080/10618600.2017.1305277 (2017).

18 Williamson, D. A. *et al.* Bridging of Neisseria gonorrhoeae lineages across sexual networks in the HIV pre-exposure prophylaxis era. *Nat Commun* **10**, 3988, doi:10.1038/s41467-019-12053-4 (2019).
