# Supplementary material for: Adaptation to the cervical environment is associated with increased antibiotic susceptibility in Neisseria gonorrhoeae
Source: Nat Commun. 2020 Aug 17;11:4126. doi: 10.1038/s41467-020-17980-1 (PMC7431566; doi:10.1038/s41467-020-17980-1)
Supplement: Supplementary file 3 — Descriptions of Additional Supplementary Files [file 41467_2020_17980_MOESM3_ESM.pdf]

Descriptions of Additional Supplementary Files

Supplementary Data 1.

Description: Multiple regression results for log-transformed antibiotic MICs comparing models with and without MtrC LOF.

Supplementary Data 2.

Description: Annotated significant unitigs from N. gonorrhoeae GWAS conducted on logtransformed antibiotic MICs.

Supplementary Data 3.

Description: SRA accession numbers, genotypes, antimicrobial phenotypes, and patient metadata for all isolates in the N. gonorrhoeae global collection.

Supplementary Data 4.

Description: SRA accession numbers, genotypes, antimicrobial phenotypes, and patient metadata for all isolates in the N. gonorrhoeae Australia validation collection.

Supplementary Data 5.

Description: PubMLST accession numbers, urogenital clade membership, and MtrC LOF status for all isolates in the ST-11 N. meningitidis collection.
