# Supplementary material for: Adaptation to the cervical environment is associated with increased antibiotic susceptibility in Neisseria gonorrhoeae
Source: Nat Commun. 2020 Aug 17;11:4126. doi: 10.1038/s41467-020-17980-1 (PMC7431566; doi:10.1038/s41467-020-17980-1)
Supplement: Supplementary file 9 — Reporting Summary [file 41467_2020_17980_MOESM9_ESM.pdf]

nature research

Corresponding author(s): Yonatan Grad

Last updated by author(s): Jul 14, 2020

Reporting Summary

Nature Research wishes to improve the reproducibility of the work that we publish. This form provides structure for consistency and transparency in reporting. For further information on Nature Research policies, see Authors & Referees and the Editorial Policy Checklist .

Statistics

For all statistical analyses, confirm that the following items are present in the figure legend, table legend, main text, or Methods section.

n/a	Confirmed	
☐	☒	The exact sample size (*n*) for each experimental group/condition, given as a discrete number and unit of measurement
☐	☒	A statement on whether measurements were taken from distinct samples or whether the same sample was measured repeatedly
☐	☒	The statistical test(s) used AND whether they are one- or two-sided<br>*Only common tests should be described solely by name; describe more complex techniques in the Methods section.*
☐	☒	A description of all covariates tested
☐	☒	A description of any assumptions or corrections, such as tests of normality and adjustment for multiple comparisons
☐	☒	A full description of the statistical parameters including central tendency (e.g. means) or other basic estimates (e.g. regression coefficient) AND variation (e.g. standard deviation) or associated estimates of uncertainty (e.g. confidence intervals)
☐	☒	For null hypothesis testing, the test statistic (e.g. *F*, *t*, *r*) with confidence intervals, effect sizes, degrees of freedom and *P* value noted<br>*Give P values as exact values whenever suitable.*
☒	☐	For Bayesian analysis, information on the choice of priors and Markov chain Monte Carlo settings
☒	☐	For hierarchical and complex designs, identification of the appropriate level for tests and full reporting of outcomes
☐	☒	Estimates of effect sizes (e.g. Cohen's *d*, Pearson's *r*), indicating how they were calculated

Our web collection on statistics for biologists contains articles on many of the points above.

Software and code

Policy information about availability of computer code

Data collection	fastq-dump in SRA toolkit version 2.8.1 was used to acquire sequencing read data
Data analysis	FastQC (version 0.11.7), BWA-MEM (version 0.7.17-r1188), Picard (version 2.8.0), BamQC (version 2.2.1), Pilon (version 1.16), SPAdes (version 3.12.0), Prokka (version 1.13), Roary (version 3.12), piggy (version 1.2), Gubbins (version 2.3.4), iTOL (version 4.4.2), BLASTn (version 2.6.0), MAFFT (version 7.450), FastBAPS (version 1.0.0), R (version 3.5.1), Pyseer (version 1.2.0), GATB (version 1.3.0), PubMLST, Python (version 3.6.5), Biopython (version 1.69), Geneious Prime (version 2019.2.1), custom code: https://github.com/gradlab/mtrC-GWAS

For manuscripts utilizing custom algorithms or software that are central to the research but not yet described in published literature, software must be made available to editors/reviewers. We strongly encourage code deposition in a community repository (e.g. GitHub). See the Nature Research guidelines for submitting code & software for further information.

Data

Policy information about availability of data

All manuscripts must include a data availability statement. This statement should provide the following information, where applicable:

- Accession codes, unique identifiers, or web links for publicly available datasets
- A list of figures that have associated raw data
- A description of any restrictions on data availability

In Supplementary Data 3-4, we have included accession numbers (via publicly hosted database NCBI SRA) for accessing all raw sequence data used for N. gonorrhoeae analyses. Intermediate outputs from the genomics pipeline (e.g., de novo assemblies) may also be available from the authors upon request. In Supplementary Data 5, we have included accession numbers (via publicly hosted database PubMLST) for accessing all sequence data used for N. meningitidis analyses. Source data underlying all figures are available in Supplementary Data 1-2 or at https://github.com/gradlab/mtrC-GWAS.

Field-specific reporting

Please select the one below that is the best fit for your research. If you are not sure, read the appropriate sections before making your selection.

☐ Life sciences ☐ Behavioural & social sciences ☒ Ecological, evolutionary & environmental sciences

For a reference copy of the document with all sections, see nature.com/documents/nr-reporting-summary-flat.pdf

Ecological, evolutionary & environmental sciences study design

All studies must disclose on these points even when the disclosure is negative.

Study description	Genome-wide association study of Neisseria gonorrhoeae followed by patient epidemiological analyses
Research sample	Genome sequencing data along with antimicrobial resistance phenotypes and associated patient epidemiological data for (in total) n=4852 strains (all publicly available - i.e., on NCBI SRA - N. gonorrhoeae genome sequences with associated metadata at time of study inclusion).
Sampling strategy	After literature search at the time of study initiation, all available studies with at least 10 isolates with antimicrobial resistance and whole-genome sequencing data were included in our meta-analysis global collection. This comprehensive sampling strategy gave us the largest number of collected gonococcal isolates to date. During the course of the study, an additional validation dataset of 2186 isolates was published. We included this to further validate our statistical associations.
Data collection	Relevant studies were identified through a systematic literature review.
Timing and spatial scale	Literature review and study assembly was conducted from August 2018 to January 2019 for the global meta-analysis collection, followed by computational and statistical analyses. These isolates spanned 65 countries collected over 38 years. Further study assembly of the validation dataset was conducted in May 2019.
Data exclusions	Data were only excluded if they failed to meet pre-established genomics quality control benchmarks stated in the methods.
Reproducibility	Observed patient epidemiological associations were successfully replicated in an independent, validation cohort of strains described in the text. This was the only validation cohort of strains for which replication was attempted. Code to replicate these analyses is available on Github. Experimental validation of GWAS findings using antimicrobial resistance testing was conducted in triplicate with nearly identical results.
Randomization	Country of origin was a possible confounder for antibiotic resistance differences between isolates, and was included as a covariate in the GWAS to account for country-level differences in MIC testing and reporting practices.
Blinding	Blinding was not relevant to this study as it was an observational study without an interventional treatment.

Did the study involve field work? ☐ Yes ☒ No

Reporting for specific materials, systems and methods

We require information from authors about some types of materials, experimental systems and methods used in many studies. Here, indicate whether each material, system or method listed is relevant to your study. If you are not sure if a list item applies to your research, read the appropriate section before selecting a response.

Materials & experimental systems

n/a	Involved in the study	
☒	☐	Antibodies
☒	☐	Eukaryotic cell lines
☒	☐	Palaeontology
☒	☐	Animals and other organisms
☒	☐	Human research participants
☒	☐	Clinical data

Methods

n/a	Involved in the study	
☒	☐	ChIP-seq
☒	☐	Flow cytometry
☒	☐	MRI-based neuroimaging
